# Structural, magnetic, and electronic properties of Fe_82_Si_4_B_10_P_4_ metallic glass

**DOI:** 10.1038/s41598-018-23952-9

**Published:** 2018-04-09

**Authors:** Hui Chen, Bangshao Dong, Shaoxiong Zhou, Xinxin Li, Jingyu Qin

**Affiliations:** 1 Advanced Technology & Materials Co., Ltd., Central Iron & Steel Research Institute, Beijing, 100081 China; 2 Key Laboratory for Liquid-Solid Structural Evolution and Processing of Materials (Ministry of Education), Shandong University, Jinan, 250061 China

## Abstract

The structural, magnetic, and electronic properties of Fe_82_Si_4_B_10_P_4_ metallic glass were systemically investigated by theoretical simulations. Strong atomic interaction between Fe and metalloid atoms can be observed, while the direct metalloid-metalloid atomic bonds are rare due to the solute-solute avoidance effect. The calculated value of saturation magnetic flux density (*B*_*S*_) is ~1.65 T, approaching to experimental result, which is contributed by not only the electron exchange between Fe and metalloid atoms, but also the *p*-*d* orbital hybridization of Fe atoms. Moreover, Fe atoms with neighboring P atom behaving larger magnetic moments reveals the important role of P element for promoting the *B*_*S*_ value. The potential correlation between magnetic behaviors and the local atomic packing in this study sheds some light on the structural origin of the soft magnetic properties and, thereby the theoretical guidance for the development of new soft-magnetic metallic glasses.

## Introduction

Fe-based metallic glasses, as one kind of soft magnetic materials, have great potential in applications on the fields of electronic information and new-energy automobile due to its cheap cost and excellent performance^1–6^. Fe rich metallic glasses, as one type of these distinct materials with increased saturation magnetic flux density (*B*_*S*_), make it possible to be utilized for electric appliances and devices, which appeals to particular scientific interest^1,7–10^. On an experimental aspect, it is known that the richer content of Fe in the alloy, the higher value of *B*_*S*_ it owns. FeSiBPCu alloys with Fe content of 85 at.% exhibit higher *B*_*S*_ of 1.8–1.9 T and lower core loss compared to the silicon steel, resulting in reduction of CO_2_ emission and contribution to energy saving^1^. FeSiBPCu amorphous alloy ribbons containing 84.3 at.% of Fe possess *B*_*S*_ of ~1.5–1.68 T and coercivity of ~41–120 Am^−1^, which is applicable to high *B*_*S*_ and frequency fields^7^.

On the other hand, it is expected to clarify explicit structural feature and magnetic property in these alloys in view of atomic scale by using the theoretical simulation^11–15^. It has been explained theoretically that the excellent magnetic properties of Fe_76_Si_9_B_10_P_5_ metallic glass originate from structural characters that B/P centered clusters surrounded by high coordinated Fe atoms and sparse Si-rich regions^11^. For Fe rich metallic glasses, it has been indicated by using *ab initio* molecular dynamics (AIMD) that Fe_85_Si_2_B_9_P_4_ requires fast annealing to attain optimum nano-crystallization due to considerably large diffusion rates of B and P caused by their low coordination numbers^12^. Therefore, the structural feature of material is of great importance to physical properties. It becomes essential to understand the relationship between the structure and physical properties of Fe rich metallic glasses with other proportion of elements. However, less attention has been paid so far.

In this work, we investigate the structural, magnetic, and electronic characters of Fe_82_Si_4_B_10_P_4_ metallic glass by means of AIMD. It is found that there is strong interaction between Fe and metalloid atoms. The dynamic behavior of four species are studied. Moreover, the calculated value of *B*_*S*_ is 1.65 T at 300 K, resulting from not only the interaction between Fe and metalloid elements but also *p-d* orbital hybridization. These results are expected to provide wide perspective in developing novel electronic devices.

## Computational Method

Fe_82_Si_4_B_10_P_4_ alloy obtained by quenching the liquid was selected for the compositional dependence of as-quenched structure and supercooled liquid region^5^. Based on limitations of experimental measurement, AIMD simulations provide an alternate possibility for understanding the short-range structure in alloys. First-principles simulations were carried out employing generalized gradient approximation (GGA)^16,17^ with Perdew-Burke-Ernzerhof (PBE) formalism on the basis of density functional theory (DFT) as implemented in the Vienna *ab initio* simulation package (VASP)^18–21^. Compared to previous literatures^14,22,23^, we found an analogy between the calculated results of Fe_82_Si_4_B_10_P_4_ containing 200 atoms and that of primitive cell with 100 atoms. In view of the huge cost of computing resources and time, the preferred cell with 100 atoms was considered during the simulation. In view of short range order of metallic glasses, 100 atoms are located in a unit cell and proceed the relaxation to obtain the disorder and close-packed structure. Automatic k-point mesh^24^ of 1 × 1 × 1 containing Gamma was used for geometry optimization. The convergence criteria used in the electronic self-consistent and ionic relaxation were set to 10^−4^ eV and 10^−3^ eV for energy, respectively. For the metallic glass, isothermal process was simulated at 300 K with 2000 steps being collected for structural analysis^25^. Electron spins were taken into account throughout the simulation.

## Results and Discussion

### Structural study of Fe_82_Si_4_B_10_P_4_ metallic glass

#### Atomic pair correlation in Fe_82_Si_4_B_10_P_4_ metallic glass

**Figure 1:**
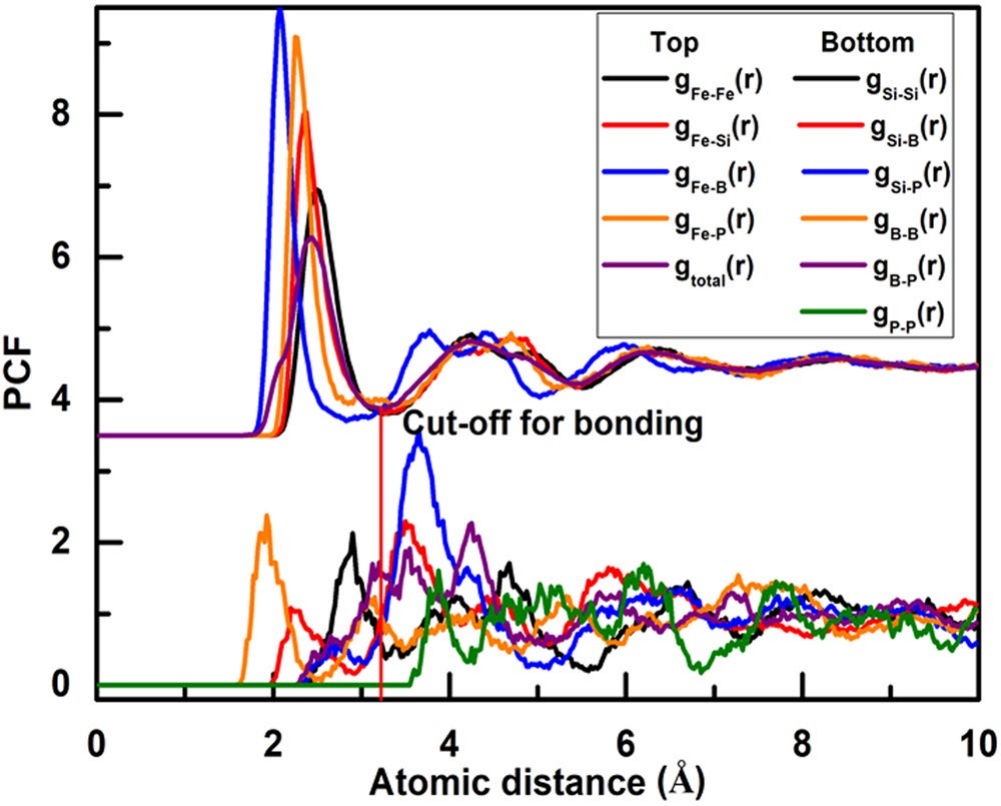
PCF in the Fe_82_Si_4_B_10_P_4_ metallic glass at 300 K.

As the first step of structural analysis, the partial and total pair correlation functions (PCFs)^26^ of Fe_82_Si_4_B_10_P_4_ are plotted in Fig. 1, which all of significant broad peaks are observed, demonstrating the appearance of amorphous structure. There is strong interaction between the Fe and metalloid (M = Si, B, and P) atoms, resulting from their intense first-nearest peaks with Fe in the *g*_Fe-M_ (*r*) curves, which is similar to Fe_76_Si_9_B_10_P_5_^14^. Due to different bonding strengths between Fe-Fe and Fe-Si pairs, the *g*_Fe-Fe_ (*r*) curve is similar to *g*_Fe-Si_ (*r*) except for a slight deviation of 0.1 Å in the first peak position, which analogous situation exists in Fe_76_Si_9_B_10_P_5_^16^. The *g*_Si-Si_ (*r*), *g*_Si-B_ (*r*), *g*_Si-P_ (*r*), and *g*_P-P_ (*r*) curves possess negligible first-nearest peaks, suggesting the presence of solute-solute avoidance^27,28^.

The primary peak of total PCF in Fig. 1, which is obtained from normalization and superposition of partial PCFs, originates from large amount of Fe-B neighboring pairs. The position of first minimal in the curve (3.2 Å) after the primary peak can be considered as the range of the nearest neighboring atoms in Voronoi polyhedron analysis. By analogy, the similar conclusions are expected to be applicable to other FeSiBP metallic glasses such as Fe_76_Si_9_B_10_P_5_ and Fe_85_Si_2_B_9_P_4_^11,12,29^.

#### Characteristics of Fe- and M-centered Voronoi clusters in Fe_82_Si_4_B_10_P_4_ metallic glass

**Figure 2:**
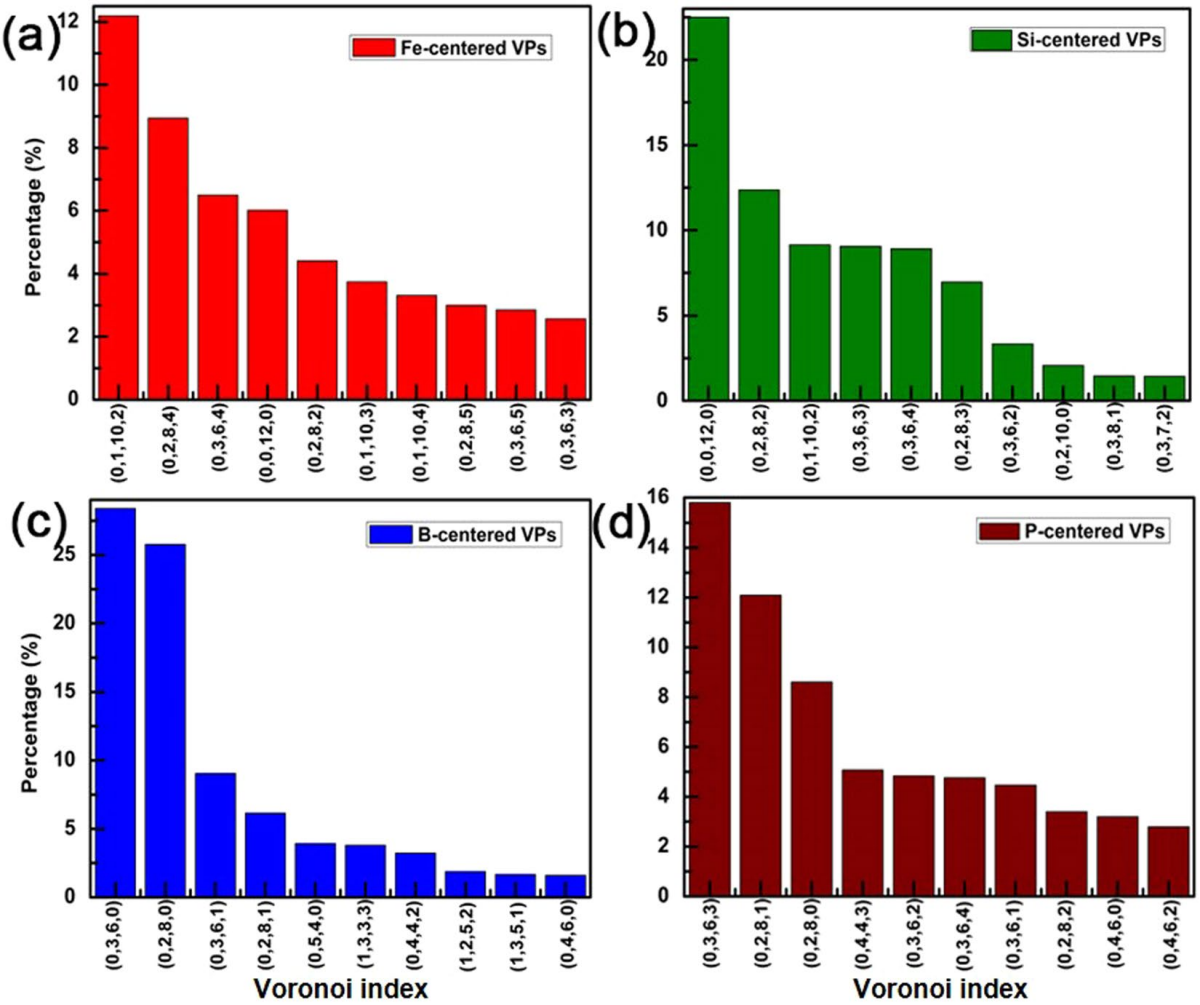
Distributions of typical Voronoi polyhedral indices of (**a**) Fe-, (**b**) Si-, (**c**) B-, and (**d**) P-centered clusters in the Fe_82_Si_4_B_10_P_4_ metallic glass at 300 K. The signature (*n*_*3*_, *n*_*4*_, *n*_*5*_, *n*_*6*_) represents Voronoi polyhedral index, in which *n*_*i*_ denotes the number of *i*-sided faces.

The Voronoi polyhedron analysis is used to simulate amorphous structure in order to determine the atomic stacking characters^30,31^. It is defined as the polyhedron with minimum volume which is constituted by the vertical bisected surfaces between the atom and its neighbor atoms. We employ the signature (*n*_*3*_, *n*_*4*_, *n*_*5*_, *n*_*6*_) to represent forms of the polyhedron, in which *n*_*i*_ denotes the number of *i*-sided faces^25^. The major types of Voronoi polyhedral indices of Fe- and M-centered clusters are shown in Fig. 2.

For Fe-centered clusters, (0, 1, 10, 2) polyhedron takes the maximum proportion, as is shown in Fig. 2a. Its derived polyhedrons such as (0, 1, 10, 3) and (0, 1, 10, 4), which occupy the similar rates, are also found around Fe atoms. All of these polyhedrons take the proportion of 19.2%. Among Si-centered Voronoi polyhedrons (Fig. 2b), (0, 0, 12, 0) takes the maximum ratio of 22.5%, indicating that icosahedron mainly exists around Si atoms. Polyhedrons (0, 3, 6, 0) and (0, 3, 6, 1) play a major role around B atoms and reach the rate of 37.4%, which suggests that tri-capped trigonal prism mostly surrounding B atoms. (0, 3, 6, 3), (0, 3, 6, 2), (0, 3, 6, 4), and (0, 3, 6, 1) polyhedrons primarily exist around P atoms, occupying the percentage of 29.9%, which suggests that P atoms are mainly surrounded by deformed tri-capped trigonal prism. These mostly conform to behavior of Fe_85_Si_2_B_9_P_4_ metallic glass^12^.

#### Fe- and M-centered coordination number in Fe_82_Si_4_B_10_P_4_ metallic glass

**Figure 3:**
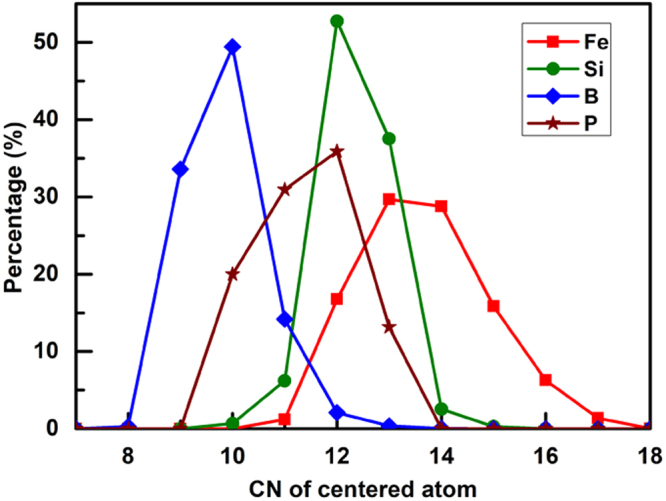
Distributions of coordination numbers in Fe- and M-centered clusters of Fe_82_Si_4_B_10_P_4_ metallic glass at 300 K.

The investigation of coordination number is in view of the basic theory of Voronoi partition^12^. The distributions of coordination numbers in Fe- and M-centered clusters are indicated in Fig. 3.

The average coordination number of Fe atom reaches the maximum of 13 among four elements listed in Table 1, which reveals that the distribution of atoms performs densely, further demonstrating that there is strong interaction between Fe and other atoms. Corresponding to each coordination number between 9 and 17, four elements possess different percentage, which proves that the distributions of size in clusters around every element are diverse. The coordination number is identical with that of Fe_85_Si_2_B_9_P_4_ metallic glass except P^12^. P in Fe_82_Si_4_B_10_P_4_ has larger coordination number by virtue of more Fe atoms located around P.

**Table 1:** Average volume occupation and coordination number of the elements in the Fe_82_Si_4_B_10_P_4_ metallic glass.

Element	Fe	Si	B	P
Volume (Å^3^)	11.05	12.00	7.85	13.33
Coordination number	13	12	10	12

#### Chemical short-range order (CSRO) of Fe- and M-centered clusters in Fe_82_Si_4_B_10_P_4_ metallic glass

**Figure 4:**
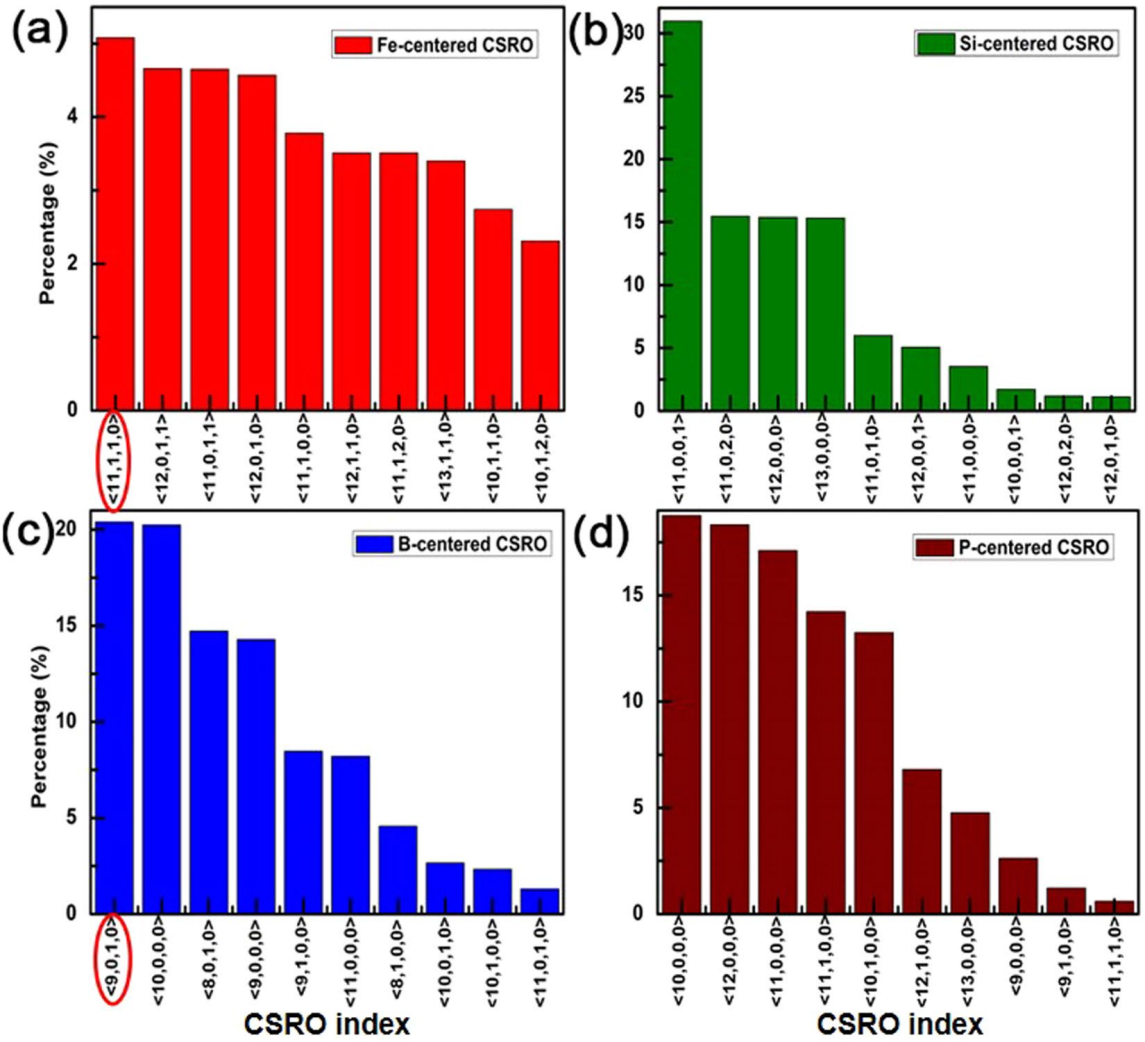
Fractions of the Fe- and M-centered CSROs in Fe_82_Si_4_B_10_P_4_ metallic glass at 300 K. The index <* n*_*1*_, *n*_*2*_, *n*_*3*_,* n*_*4*_ > represents the CSRO of clusters, in which *n*_*i*_ indicates the atomic number of Fe, Si, B, and P respectively in the chemical component of clusters. The largest fraction among S-type CSROs is labelled by a red ellipse.

The fractions of Fe- and M-centered CSROs are shown in Fig. 4 and Table 2. The index < *n*_*1*_, *n*_*2*_, *n*_*3*_, *n*_*4*_ > is considered as the CSRO type, in which *n*_*i*_ indicates the atomic number in the chemical component^32^. For instance, index < 11, 1, 1, 0 > in Fig. 4a denotes that the Fe-centered CSRO in Fe_82_Si_4_B_10_P_4_ contains 11 Fe atoms, 1 Si atom, 1 B atom, and 0 P atom. We define CSRO in which the nearest neighbor (NN) atoms are all Fe as P-type and consider M-centered CSRO in which other same M atom exists as S-type^14^.

**Table 2:** Type and fraction of atom-centered CSROs in Fe_82_Si_4_B_10_P_4_ metallic glass at 300 K.

Alloy	CSRO type	Fe-	Si-	B-	P-
Fe_82_Si_4_B_10_P_4_	P-type	0	34.8%	43.5%	61.7%
	S-type	100%	0	46.9%	0

It is seen in Table 2 that the same atoms are found in NN atoms of B, which concludes that M-centered clusters appear in the Fe_82_Si_4_B_10_P_4_ metallic glass and B atoms are unable to disperse thoroughly. The same atoms are hardly found around Si or P atoms, resulting in the improving possibility of surrounding by Fe, which implies that Si and P atoms distribute dispersely and the avoidance effect exists apparently in them. For P-centered CSRO, the ratio of P-type reaches the maximum of 61.7%, indicating that Fe favors to distribute in P-centered clusters, which leads to the stronger attractive interaction between Fe and P atoms compared to other M atoms. On the other hand, Fe-centered clusters which are surrounded only by Fe are hardly found, while Fe atoms mostly are located in NN atoms of all elements, which causes uniform distributions of Fe.

#### Dynamic study of the atoms in Fe_82_Si_4_B_10_P_4_ metallic glass

**Figure 5:**
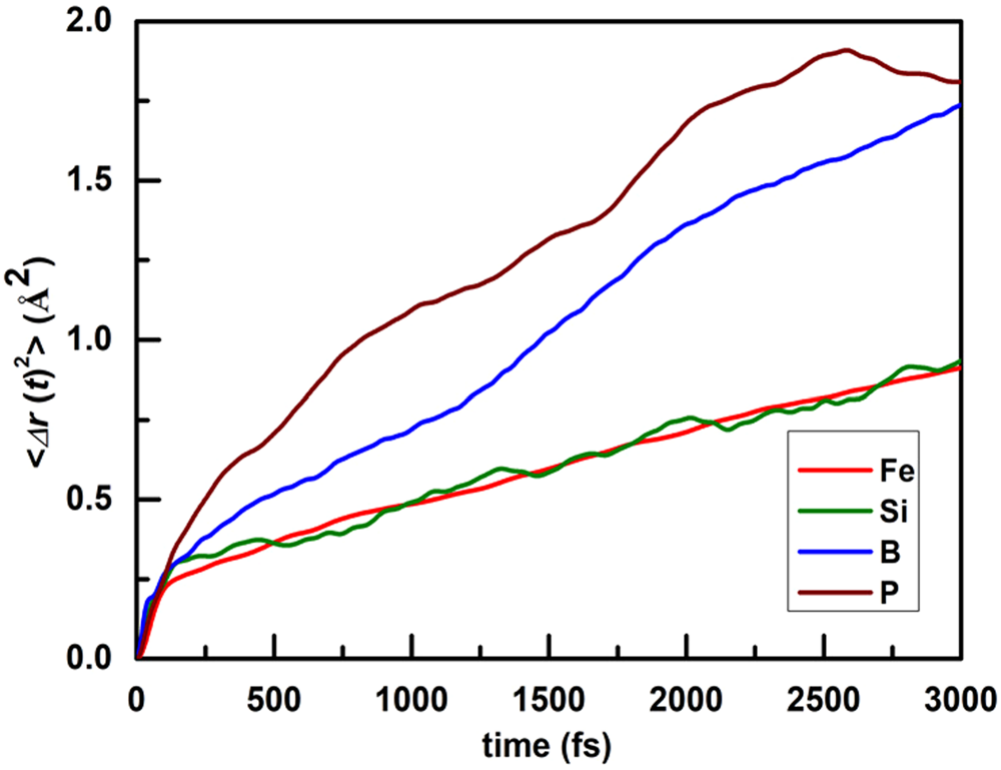
The initial step of the MSD of Fe, Si, B, and P atoms.

The crystallization process depends on not only the structural characteristic of clusters but also the diffusion ability of the atoms. The time-dependent mean-square displacement (MSD) of diffusing liquids behaves linear to t for long time^26^. Accordingly, diffusion rates of the atoms in the alloy are discussed by using MSD of the atoms which can be expressed as^33^

$$ < {\rm{\Delta }}r{(t)}^{2} > \to 6{D}_{\alpha }t+{B}_{\alpha }$$

where *Δr*(*t*)^2^ is time dependent MSD; *D*_*α*_ is the diffusion coefficient of *α* species; while *B*_*α*_ is a constant. It is evident that diffusion rates of the atoms in the alloy are proportional to the fitting slopes in MSD-t curves of the atoms. Taking into account the stronger migration ability of the atom at higher temperature, we investigate the diffusion effects at 1300 K. Figure 5 shows the MSD of Fe, Si, B, and P atoms before 3000 fs. There are minima at about 125 fs for all kinds of atoms. It is seen that the diffusion effect of P performs most strongly among these atoms owing to its enough abundance in free volumes (Table 1). On the other hand, the diffusion ability of B exceeds that of Fe and Si due to its strongest atomic activation, which results from the simulated result that the bonding energy of B (8 eV/atom) is smaller compared with other elements (~9 eV/atom). Therefore, the migration ability of an atom depends on not only free space of its movement but also energetic activation to damage the bonding between its neighbors and it. In addition, there is little difference in the diffusion coefficient between Fe and Si, which shows the existence of drastic cooperative action. Taking into account of the dynamic performance at higher temperature of 1800 K, the diffusion ability of B surpasses that of P by virtue of its strongest energetic activation, which is similar to Fe_85_Si_2_B_9_P_4_ metallic glass at 1800 K^12^.

#### Magnetic study of Fe_82_Si_4_B_10_P_4_ metallic glass

To study the magnetic property of the material, the electrons’ spins were considered during the simulation. Total magnetic moment of Fe_82_Si_4_B_10_P_4_ becomes stable at 130 *μ*_B_ at 300 K. According to the relationship between *B*_*S*_ and magnetic moment (*M*) involving supercell volume (*V*_*S*_) *B*_*S*_ = *μ*_0_(*M*/*V*_S_), where *μ*_0_ is vacuum permeability (4π × 10^−7^ TmA^−1^), the calculated value of *V*_*S*_ is 918.6 Å^3^ and then that of *B*_*S*_ is 1.65 T, which is slightly below experimental result of Fe_82_Si_4_B_11_P_3_ (1.66 T)^32^. This relates to their different contents of B and P.

**Figure 6:**
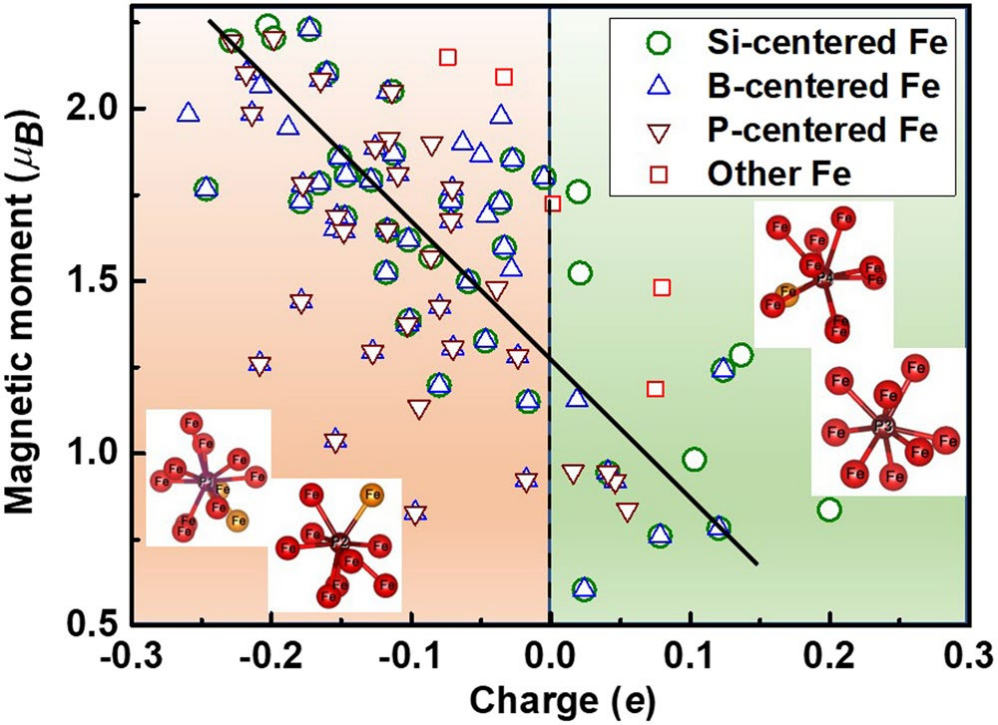
The relationship between magnetic moments and electron charges of Fe atoms in Fe_82_Si_4_B_10_P_4_ metallic glass. The inset pictrues are the configurations of P and its NN Fe atoms, in which orange balls indicate four Fe atoms obtaining electrons.

The average quantities and distributions of charges in every element of this alloy from Bader analysis^34^ are listed in Table 3. From the average excess charge, the abilities of B and P acquiring electrons (~0.5 *e*) from neighboring atoms are stronger than that of Fe and Si losing electrons (~0.1 *e*), relating to the tendency of their transition to electric neutrality and their stronger atomic activation. It is clarified that the charge distribution of Fe is more extensive than other elements even if electrons are distributed irregularly, which illustrates that Fe atoms play a major role in electron transfer. In order to distinguish the cause of enhancing *M* and *B*_*S*_, the relationship between local magnetic moment of Fe and amount of electron charge is described in Fig. 6. It is obvious that Fe with less electron charge possesses larger magnetic moment, namely, while Fe losing more electrons is provided with larger magnetic moment, the magnetization of those obtaining more electrons exhibit weaker. All Fe atoms are classified four types, such as the first NN Fe of Si, B, and P and other Fe atoms, which is marked in Fig. 6. The position of first minimal in the curve of Fig. 1 (3 Å) after the primary peak can be considered as the range of NN Fe atoms centered by Si, B, and P. While Fe atoms around P mainly focus in the region of lost electrons and larger magnetic moments, those around Si and B with weaker magnetization acquiring electrons are more than P. It reveals the truth that the charge transfer between Fe and metalloid elements enable the free electrons of Fe to excite to high spin state and strengthen overall magnetic moment. Moreover, the supplement of P improves the ability of Fe losing electrons due to strong attractive interaction between them, leading to more exited electrons, which results in superior soft-magnetic characteristics and glass formation ability to corresponding character of FeSiB alloys^32^. Meanwhile, four NN Fe atoms of P obtain 0.02*e*, 0.04*e*, 0.05*e*, and 0.06*e* from their neighboring Fe and Si atoms, respectively, which results from stronger interaction between their ambient Fe/Si and them. In the view of electron orbital, it is shown from the partial magnetic moments in Table 3 that Fe, Si, B, and P spin polarizations mainly originate from 4*d* and *p* states, which causes by *p-d* orbital hybridization. While the average positive magnetic moment of Fe is 1.550 *μ*_*B*_, B possesses the largest negative spin polarization of −0.099 *μ*_*B*_, which implies that B weakens the magnetic property to a greater extent in contrast with Si and P.

**Table 3:** Valence charge, magnetic moment of *s* (*m*_*s*_), *p* (*m*_*p*_), *d* (*m*_*d*_), and total (*m*) orbitals for the atoms in the Fe_82_Si_4_B_10_P_4_ metallic glass.

Element	Fe	Si	B	P
Distribution (Range) of charge (*e*)	7.74~8.20 (0.46)	3.74~4.07 (0.33)	3.38~3.56 (0.18)	5.52~5.59 (0.07)
Average charge (*e*)	7.92	3.89	3.47	5.56
Average excess charge (*e*)	−0.08	−0.11	0.47	0.56
Distribution (Range) of *m* (*μ*_*B*_)	0.604~2.24 (1.64)	−0.059~−0.073 (0.014)	−0.078~−0.118 (0.04)	−0.043~−0.061 (0.018)
Average *m*_*s*_ (*μ*_*B*_)	−0.007	−0.010	−0.017	−0.003
Average *m*_*p*_ (*μ*_*B*_)	−0.023	−0.057	−0.081	−0.052
Average *m*_*d*_ (*μ*_*B*_)	1.583	—	—	—
Average *m* (*μ*_*B*_)	1.550	−0.067	−0.099	−0.054

#### Electronic density of states of Fe_82_Si_4_B_10_P_4_ metallic glass

**Figure 7:**
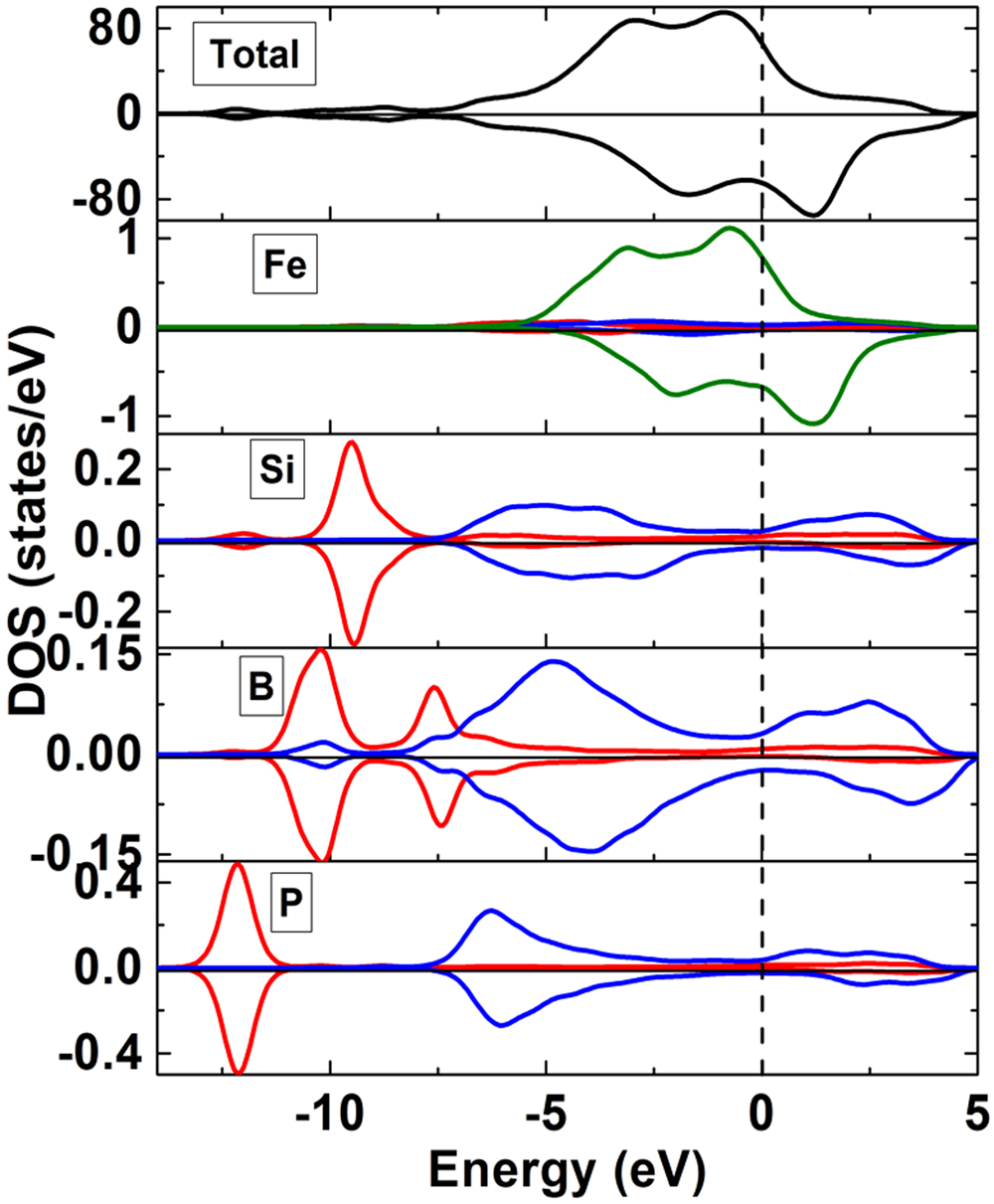
Total and partial density of states in Fe_82_Si_4_B_10_P_4_ metallic glass. The red, blue, and green curves represent the *s*,* p*, and *d* orbitals, respectively. The vertical dashed lines denote the Fermi level and are set to zero.

We focus our attention on the density of states (DOS)^35^ of the metallic glass calculated using a 2 × 2 × 2 k-point mesh, as is shown in Fig. 7. It has been verified that strong ferromagnetism is found when the Fermi level (*E*_*F*_) located in the gap of spin-down bands^36,37^. Accordingly, Fe_82_Si_4_B_10_P_4_ metallic glass behaves ferromagnetic. The bands near Fermi level (*E*_*F*_) are mainly attributed to Fe-3*d* states with a small contribution from p states of Si, B, and P, manifesting evidently the hybridization between *p* and *d* orbitals. The energy above *E*_*F*_ stems from the hybridization between Fe-3d and B-2p states with a small contribution of 3*p* states of Si and P. The bands between −7.5 eV and −5 eV below *E*_*F*_ are mainly contributed by the hybridization of Si-3*p*, B-2*p*, and P-3*p* orbitals as well as a small amount of B-2*s* states. The lower bands in the range from −11 eV to −7.5 eV are primarily from Si-3*s* and B-2*s* states. The P-3*s* states are situated at much deeper energy around −12.5 eV, as shown in the lowest plane of Fig. 7.

## Conclusions

The structural, magnetic, and electronic characters of the Fe_82_Si_4_B_10_P_4_ metallic glass have been discussed using AIMD. Among four elements, there is strong interaction between Fe and metalloid atoms while solute-solute avoidance exists in metalloid atoms, especially manifesting obviously in Si- and P-centered clusters. P has superior migration ability to other elements owing to its enough abundance in free volumes. The value of *B*_*S*_ reaches 1.65 T at 300 K approximating to experimental result. Its high *B*_*S*_ stems from not only charge exchange between metalloid atoms and Fe but also pd orbital hybridization. The nearest neighboring Fe atoms around P mainly lose electrons and possess larger magnetic moments owing to strong attractive interaction between them. From the angle of atomic orbitals, the spin polarizations of Fe and metalloid atoms mainly originate from 3*d* and *p* states, respectively. It is further illustrated from the electronic density of states the hybridization between 3*d* states of Fe and *p* states of metalloid atoms at Fermi level. These offer theoretical references for exploiting novel energy-efficient and environment-friendly soft-magnetic materials.
